# Brucellosis with rare complications and review of diagnostic tests: a case report

**DOI:** 10.1186/s13256-022-03702-2

**Published:** 2022-12-30

**Authors:** Arzu Altunçekiç Yildirim, Celali Kurt, Yeliz Çetinkol

**Affiliations:** 1 Department of Infectious Diseases and Clinical Microbiology, Faculty of Medicine, Ordu University, Ordu, Turkey; 2 Department of Medical Microbiology, Faculty of Medicine, Ordu University, BucakMah. NefsiBucak Cad. 52200, Ordu, Turkey

**Keywords:** Brucellosis, Spondylodiscitis, Paravertebral abscess, Empyema

## Abstract

**Background:**

Brucellosis is one of the most common zoonotic diseases in the world. Osteoarticular complications, especially vertebral system involvement, are most commonly reported. However, reports and coreports of pulmonary complications and thoracal spondylodiscitis and epidural abscess are rare.

**Case presentation:**

Spondylodiscitis was detected at the T11–12 vertebral level, followed by epidural and paravertebral abscess, and then empyema was detected in a 17-year-old Asian female patient without any additional disease. The patient had used various antibiotics and the disease could not be proven bacteriologically. Also, the Rose Bengal test was negative. However, serologically high titer *Brucella* positivity was detected in the blood and pleural fluid sample. Drainage was required for bilateral empyema. Disease duration prolonged due to multiple complications. The patient was cured with combined long-term treatment for brucellosis.

**Conclusions:**

Although some are rare, brucellosis is a zoonotic disease that can cause many complications. The gold standard for diagnosis is the growth of bacteria in blood culture or tissue culture. However, isolation of the microorganism can be very difficult. Clinical suspicion and serological tests are important guides.

## Background

Brucellosis is a common global zoonotic disease caused by bacteria of the genus *Brucella* and can progress with various clinical manifestations. It may result in long-term illness and various complications due to being undiagnosed and having inadequate treatments when it is not considered as a diagnosis. The disease is an important public health problem worldwide and is common in many developing countries, especially in the Mediterranean Basin, Northern and Eastern Africa, the Middle East, and the Arabian Peninsula. According to World Health Organization reports, more than 500,000 cases of brucellosis are reported annually, especially from developing countries, and it is estimated that there are four undetected cases for every diagnosed case [1]. Our country is endemic in terms of the disease, and the continuation of traditional farming practices, lifestyle, and consumption of fresh dairy products contribute to the high incidence of brucellosis. There are reports between rates of 1.3–26.7% in seroprevalence studies, and the highest rates are found mainly in the Southeastern Anatolian provinces [2].

Brucellosis, which has a clinical variety, can present with many system-related complications. Osteoarticular complications (10–85%), especially vertebral system involvement, are most commonly reported [3]. Brucellar spondylitis and spondylodiscitis mostly affect the lumbar region (60%), less frequently the thoracic (19%), and rarely the cervical segments (12%). Spinal epidural abscess is a more severe clinical form that develops secondary to spondylodiscitis. Its frequency has been reported as 1/10,000, and few cases exist in the literature [4]. The spread of epidural abscesses to the paravertebral area is much less common [5].

Pulmonary brucellosis, on the other hand, is a very rare complication due to improved diagnostic tests and treatment availability. Its incidence is 1–10% in the literature [6]. Parenchymal nodules, lobar pneumonia, or pleural effusion are among the pulmonary pathologies detected. Empyema reporting is limited to a few cases [7].

In our case report, we present a case of a 17-year-old patient with spondylodiscitis and paravertebral abscess affecting the lower thoracic vertebrae as well as brucellosis with bilateral empyema secondary to abscess without pneumonic involvement, and we share the serological tests used in the diagnosis of the case and the difficulties we experienced with these tests.

## Case presentation

**Fig. 1:**
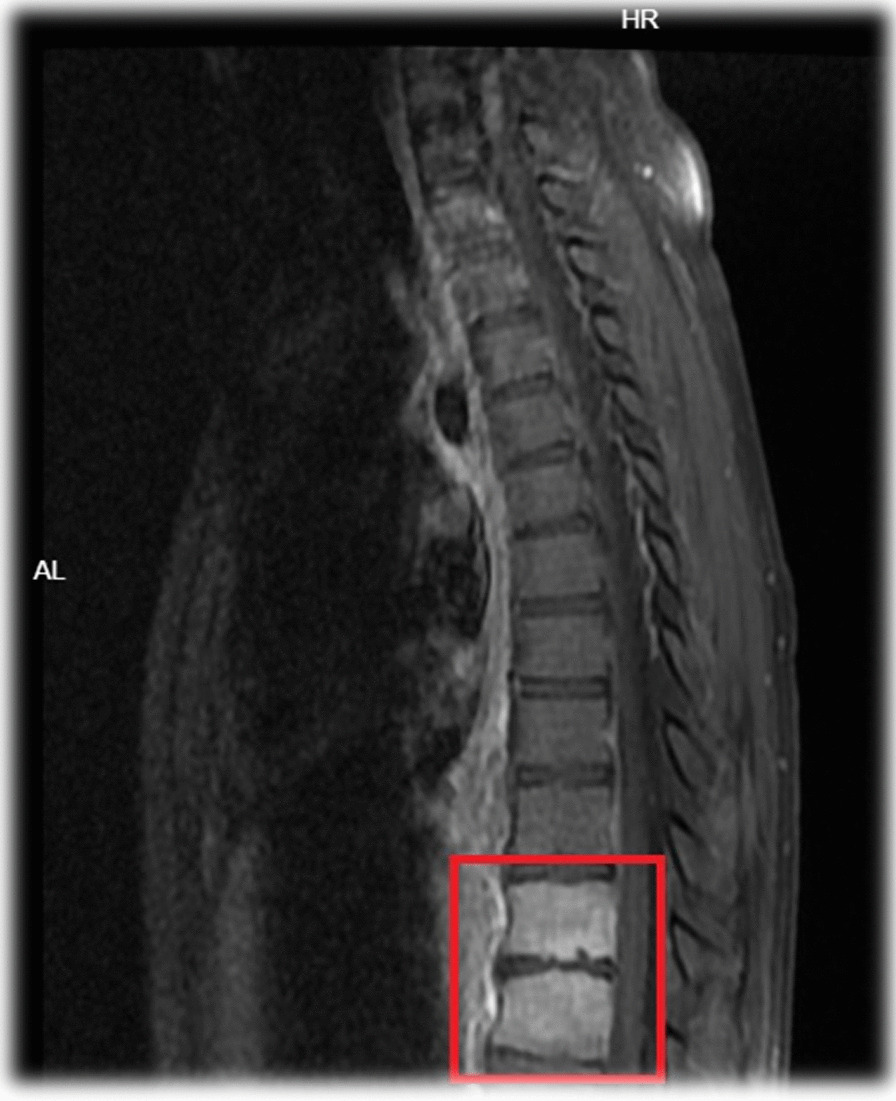
T11-12 spondylodiscitis (affected vertebrae are shown in red box)

**Fig. 2:**
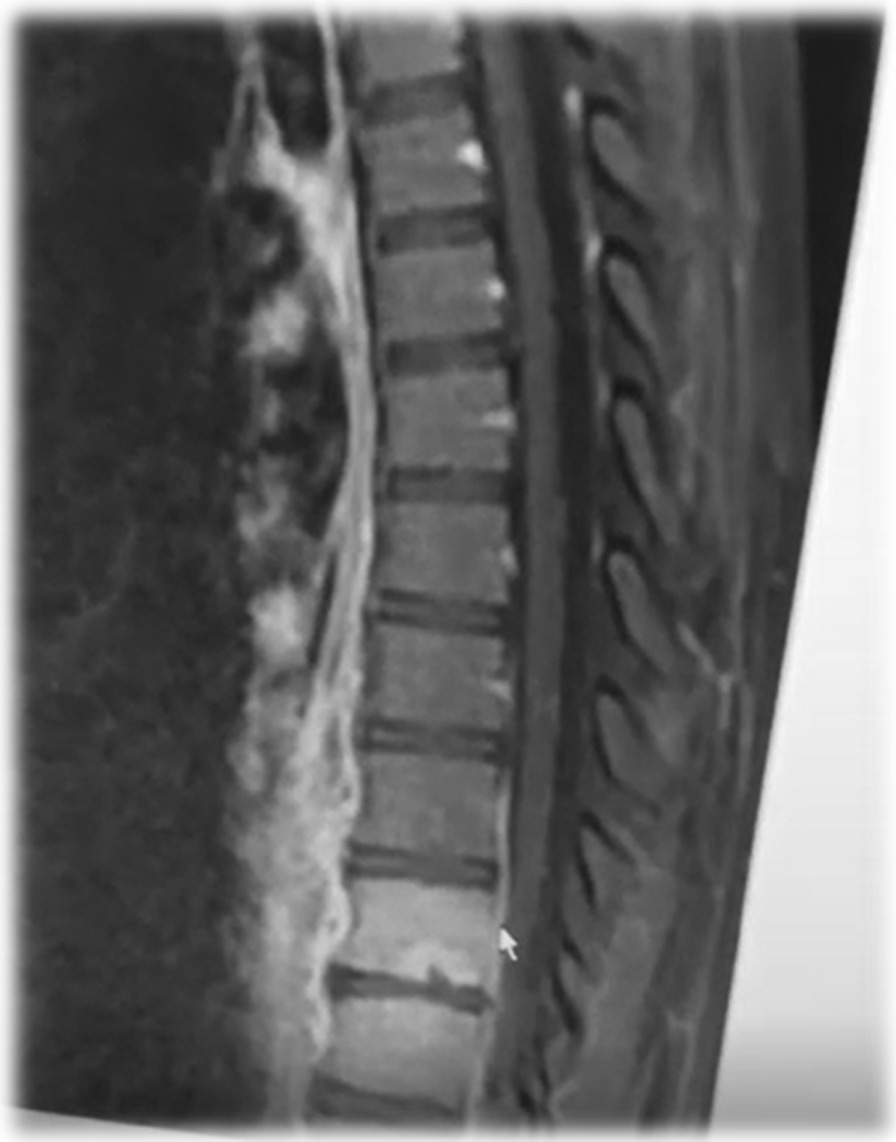
Epidural abscess and paravertebral abscess

**Fig. 3:**
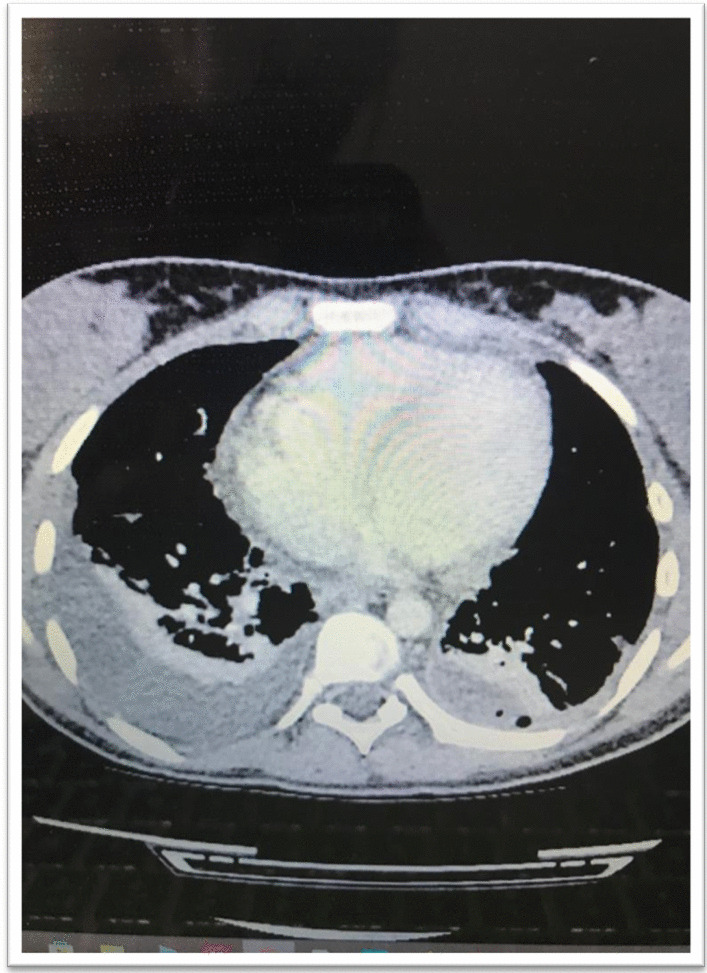
Thoracic computed tomography, mediastinal window: pleural fluid with dense content

**Fig. 4:**
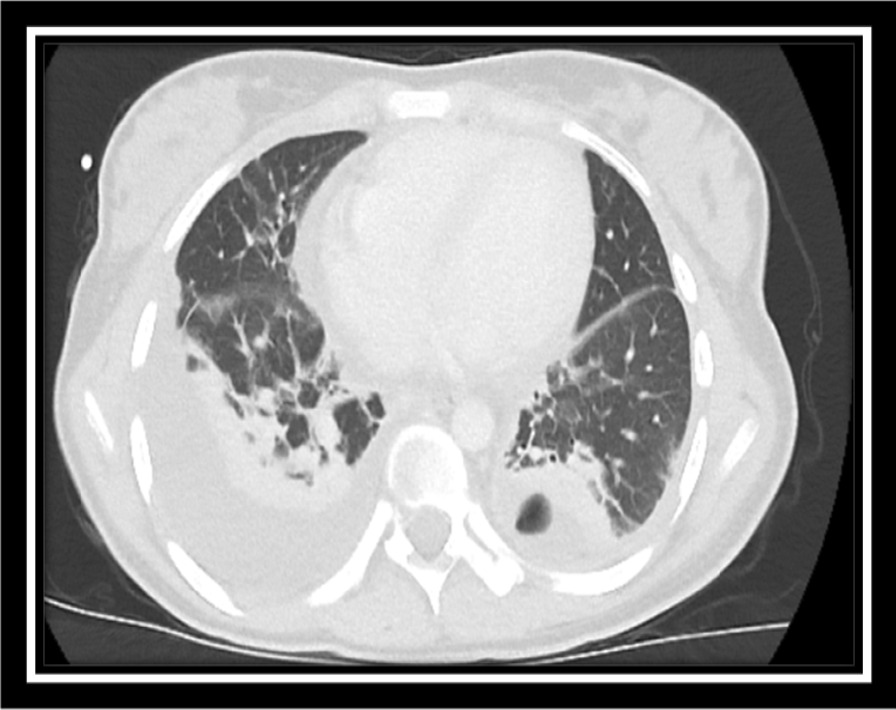
Thoracic computed tomography, lung window: pleural fluid with dense content

**Fig. 5:**
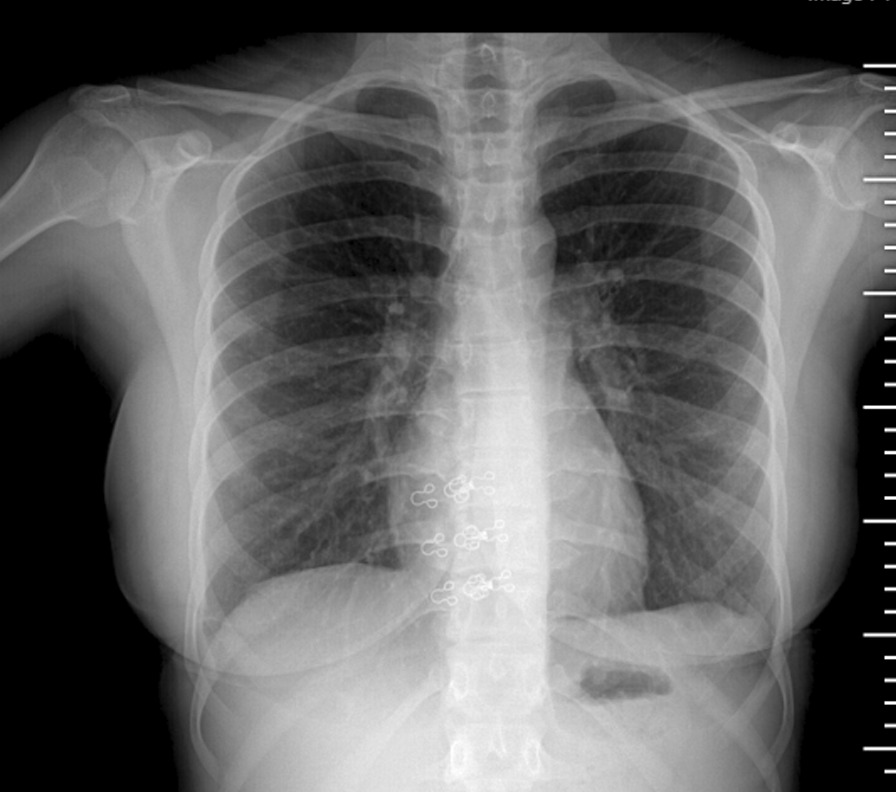
End-of-treatment lung X-ray

A 17-year-old Asian female patient living in a rural area and whose family is engaged in animal husbandry had abdominal pain, lower back pain, and increased fever, especially at night, which started approximately 1 month before the admission to our emergency department. She had no history of disease and drug use. There was no chronic disease in her family. She went to different polyclinic branches in many hospitals with these complaints. During this time, in these sections, after receiving various diagnoses and treatments such as urinary infection and lumbar discopathy, she was diagnosed at another center with brucellosis after her standard tube agglutination test (STA) was positive at 1/320 titer, and her treatment was arranged in the form of a combination of doxycycline (100 mg; twice a day) and rifampicin (600 mg; once a day). Although the treatment was given to the patient, her complaints continued, and her lower back pain worsened, so she came to our emergency department and was consulted by the department of infectious diseases. On admission, her fever was 37.8 °C, and other vital signs were stable. On physical examination, diffuse tenderness was detected on palpation in the thoracolumbar vertebrae. Other physical examination findings were normal. In the examinations performed, hemoglobin (Hb) level was 10.4 g/dL, white blood cell count was 10,900 (53% neutrophils, 35% lymphocytes), C-reactive protein (CRP) was 92.9 mg/L (0–5 mg/L), erythrocyte sedimentation rate was 71 mm/hour, Rose Bengal plate test (RBPT) was negative, and posteroanterior (PA) chest X-ray was found normal. The brucellosis treatment the patient was taking was continued. Blood cultures were taken after admission. Although the lumbar magnetic resonance (MR) imaging was normal, on contrast-enhanced thoracic vertebra MR examination, bone marrow edema in T11 and T12 vertebrae in this disc space, mild hyperintense signal change in T2W disc space on contrast-enhanced examination, and intense enhancement of the described vertebrae were observed. Spondylodiscitis was considered. At the same time, a T1W hypointense, T2W hyperintense intense peripheral enhanced signal area was detected at this level, reaching a thickness of approximately 2 mm, which may be compatible with epidural abscess and paravertebral abscess in the anterior paravertebral area (Figs. 1, 2). Ceftriaxone (1000 mg; twice a day) was added to the treatment that the patient was taking and continued. She needed serious analgesics due to severe pain. Surgical intervention was not considered for the patient by the department of neurosurgery since there was no neurological finding on physical examination. Our request for interventional sampling was not accepted. The *Brucella* agglutination test with Coombs performed in our microbiology laboratory was positive at a titer of > 1/1280. There was no contact history regarding tuberculosis, and the purified protein derivative (PPD) test was negative. No growth was detected in blood cultures during and after this period. The patient, who did not have a fever after hospitalization, complained of a fever reaching 39 °C on the fourth day. On the days following the development of fever, the control white blood cell value was 21,000, and CRP was 420 mg/L; the possibilities for another focus of infection, progression of the current complication, or another complication were evaluated. As a result of the examinations, empyema was detected in the left lung, transferred to the department of thoracic surgery, and tube thoracostomy (closed underwater drainage) was performed. An exudate-like fluid was detected with drainage and a hemopurulent appearance. On gram examination, there were plenty of leukocytes, and no microorganisms were seen. There was no growth in the pleural fluid culture. The data obtained during this intervention were limited due to insufficient examination requests. The patient’s treatment was continued during this period by changing with doxycycline, trimethoprim/sulfamethoxazole (TMP/SXT) (10 mg TMP/kg/day intravenously divided every 6–12 hours). After the necessity of tube thoracostomy disappeared, the patient was taken back to our service to continue the treatment. On the eighth day following the drainage, the patient developed a fever again, and the acute phase values increased. Thoracic computed tomography (CT) was performed by taking a blood culture. Pleural fluid appearance with dense content, reaching a thickness of 3 cm in the thickest part on the right and 25 mm in the thickest part on the left, and containing air values on the left; pleural fluid with a thickness of approximately 1 cm between the leaves of the mediastinal pleura in the right paracardiac area, the largest in the paracardiac and prevascular areas in the mediastinum; and a few lymph nodes with a short diameter of 7 mm were detected (Figs. 3, 4). Before thoracic surgery, a thoracentesis was performed, and we planned microbiological tests. In the pleural fluid examination taken by thoracentesis, the appearance was hemorrhagic, leukocyte count was 15,000/mm^3^, pH was 7.0, protein was 3.5 g/dL, pleural fluid/serum protein was 0.61 (3.5/5.7), pleural fluid/serum lactate dehydrogenase (LDH) was 8.61 (2534/256), and glucose was 50 mg/dL. In Gram staining, 75% of polymorphonuclear leukocytes (PMNL) and bacteria were not seen. Acid Resistant Bacillus (ARB) was negative, and *Mycobacterium tuberculosis* polymerase chain reaction (PCR) was negative. No growth in culture for *Mycobacterium tuberculosis* (result learned later). Pathology result reported “mixed inflammatory cell infiltration, mesothelial cell groups; it was reported as pleural effusion fluid; no neoplastic cells were found.” When the *Brucella* agglutination test with Coombs was found to be positive > 1/1280 in the pleural fluid, it was understood that the present empyema was also due to brucellosis. Despite the long incubation period, no growth was detected in blood cultures and pleural fluid samples. The patient, whose medical treatment was continued by providing drainage of the existing empyema, was discharged in a stable condition after approximately 1 month of clinical follow-up. The brucellosis treatment of the patient was completed after 6 months with the triple combination of TMP/SXT, rifampicin, and doxycycline. No additional problems were encountered in the outpatient clinic controls (Fig. 5).

## Discussion and conclusions

Vertebral osteomyelitis, spondylodiscitis, and epidural abscess are clinical pictures with an incidence of 2.4/100,000/year, which increases with age. Many patients have at least one of the risk factors, such as diabetes, immunosuppression, malignancy, or spinal surgery history. Vertebral infection should be suspected clinically with insidious back pain that increases significantly at night, accompanying fever with a rate of 35–60% and focal neurological symptoms [8]. Bacteria are generally responsible for the etiology, and *Staphylococcus aureus* (32–67%) is the most frequent finding [9]. *Coagulase-negative staphylococci*, *E. coli*, and *Pseudomonas* species may also be responsible, especially in those with a history of injecting drug use. In countries where tuberculosis and brucellosis are endemic, *Mycobacterium tuberculosis* and *Brucella* species should be considered causative agents [10]. Microbiological methods should prove diagnosis supported by imaging methods, and microbiological diagnosis of the samples taken by interventional procedures in patients without surgical indication is essential in the correct diagnosis and management of the patient. Thanks to the serological test results, a connection was established with the vertebral complication in our case. However, the development of paravertebral abscesses and bilateral empyema, which are rare complications, necessitated the differential diagnosis of other diseases, especially tuberculosis. A definitive diagnosis could only be made due to the evaluation of pleural fluid samples with microbiological examinations*.* The diagnosis can be made when *Brucella* species are isolated from vertebral, paravertebral, or epidural tissue samples, abscess material, or blood cultures or when RBPT positivity and a *Brucella* standard tube agglutination titer above 1/160 are detected in addition to clinical findings. Owing to the specificity of laboratory evaluation, only 5% of patients with suspected vertebral brucellosis require a biopsy [11].

RBPT is often used as a rapid screening test to diagnose brucellosis. Although the sensitivity of RBPT has been reported to be very high, its specificity may be low, especially in endemic areas and chronic cases [12]. The test’s positive predictive value is low and positive results must be confirmed by a more specific test. In the presence of a high antibody titer, false negativity may result due to the prozone phenomenon [13]. Therefore, RBPT or STA negativity does not exclude *Brucella* infection. Negative results can be detected in the early stages of the disease and in the presence of blocking antibodies. In clinically compatible patients, dilution of patient serum at high titers increases the sensitivity of the SAT-Coombs combination [14]. Coombs test (antihuman globulin test), which is necessary for complicated and chronic cases, eliminates the effects of blocking antibodies. Evidence obtained in a limited number of these cases reports that the Coombs test is the best indicator of relapses and seroconversion in parallel with treatment [13, 15].

BrucellaCapt test is also an immuno-agglutination technique. It is a modification of the Coombs test to detect incomplete or blocking IgG and IgA antibodies. It has similar sensitivity and specificity to classical tests in diagnosing brucellosis, and its ease of application is an important advantage [16–18].

The gold standard for diagnosis is the growth of bacteria in blood cultures or tissue cultures (for example, joint fluid or bone marrow). However, the isolation of the microorganism is complicated. Bone marrow aspirate culture is invasive and clinically impractical. The sensitivity of blood cultures is reported to be between 17% and 85% [19].

In our case, although STA was positive at a titer of 1/320 and RBPT was negative, *Brucella* agglutination test with Coombs was found to be > 1/1280 positive. Ordu province is endemic to brucellosis, and the interpretation of serological test results can be difficult. Therefore, it may be necessary to confirm negative results in both positive and suspicious patients.

It should be taken into account that negative RBPT results may be observed, especially in cases with high clinical suspicion, and may need to be supported by other serological diagnostic tests when necessary. We think that using the BrucellaCapt test is valuable in faster diagnosis in endemic areas and effective patient management. Despite the confusing clinical and laboratory results in this case, in which more than one rare complication developed, it was possible to reach the diagnosis by applying adequate and correct microbiological examinations.

## Data Availability

Data supporting the findings of this study are not publicly available but available from the corresponding author upon reasonable request.

## Abbreviations

STA: Standard tube agglutination test
Hb: Hemoglobin
CRP: C-reactive protein
ESR: Erythrocyte sedimentation rate
RBPT: Rose Bengal plate test
PA: Posteroanterior
MR: Magnetic resonance
TMP/SXT: Trimethoprim/sulfamethoxazole
CT: Computed tomography
PCR: Polymerase chain reaction
